# Metformin normalizes mitochondrial function to delay astrocyte senescence in a mouse model of Parkinson’s disease through Mfn2-cGAS signaling

**DOI:** 10.1186/s12974-024-03072-0

**Published:** 2024-04-02

**Authors:** Min Wang, Tian Tian, Hong Zhou, Si-Yuan Jiang, Ying-Ying Jiao, Zhu Zhu, Jiang Xia, Jian-Hua Ma, Ren-Hong Du

**Affiliations:** 1 Department of Geriatrics, Affiliated Brain Hospital of Nanjing Medical University, Nanjing, 210029 Jiangsu People’s Republic of China; 2 Jiangsu Key Laboratory of Neurodegeneration, Department of Pharmacology, Nanjing Medical University, Nanjing, 211166 Jiangsu People’s Republic of China; 3 Department of Endocrinology, Nanjing First Hospital, Nanjing Medical University, Nanjing, 210000 Jiangsu People’s Republic of China; 4 Department of Orthopedics, Shanghai Tongji Hospital, School of Medicine, Tongji University, Shanghai, 200065 People’s Republic of China; 5 National Demonstration Center for Experimental Basic Medical Education, Nanjing Medical University, Nanjing, 211166 Jiangsu People’s Republic of China

**Keywords:** Metformin, Astrocyte senescence, Parkinson’s disease, Mitofusin 2, cGAS-STING

## Abstract

**Background:**

Senescent astrocytes play crucial roles in age-associated neurodegenerative diseases, including Parkinson’s disease (PD). Metformin, a drug widely used for treating diabetes, exerts longevity effects and neuroprotective activities. However, its effect on astrocyte senescence in PD remains to be defined.

**Methods:**

Long culture-induced replicative senescence model and 1-methyl-4-phenylpyridinium/α-synuclein aggregate-induced premature senescence model, and a mouse model of PD were used to investigate the effect of metformin on astrocyte senescence in vivo and in vitro. Immunofluorescence staining and flow cytometric analyses were performed to evaluate the mitochondrial function. We stereotactically injected AAV carrying GFAP-promoter-cGAS-shRNA to mouse substantia nigra pars compacta regions to specifically reduce astrocytic cGAS expression to clarify the potential molecular mechanism by which metformin inhibited the astrocyte senescence in PD.

**Results:**

We showed that metformin inhibited the astrocyte senescence in vitro and in PD mice. Mechanistically, metformin normalized mitochondrial function to reduce mitochondrial DNA release through mitofusin 2 (Mfn2), leading to inactivation of cGAS-STING, which delayed astrocyte senescence and prevented neurodegeneration. Mfn2 overexpression in astrocytes reversed the inhibitory role of metformin in cGAS-STING activation and astrocyte senescence. More importantly, metformin ameliorated dopamine neuron injury and behavioral deficits in mice by reducing the accumulation of senescent astrocytes via inhibition of astrocytic cGAS activation. Deletion of astrocytic cGAS abolished the suppressive effects of metformin on astrocyte senescence and neurodegeneration.

**Conclusions:**

This work reveals that metformin delays astrocyte senescence via inhibiting astrocytic Mfn2-cGAS activation and suggest that metformin is a promising therapeutic agent for age-associated neurodegenerative diseases.

**Supplementary Information:**

The online version contains supplementary material available at 10.1186/s12974-024-03072-0.

## Background

Parkinson’s disease (PD) is the second most common age-associated neurodegenerative disease [1]. Although the pathogenesis of PD is still unclear, accumulating evidence indicates that astrocyte senescence has been proved to play a detrimental role in PD progression [2, 3]. Senescent astrocytes show large morphology and exhibit enhanced senescence marker p16 expression, elevated senescence-associated β-galactosidase (SA-β-Gal) activity, and increased senescence-associated secretory phenotype (SASP) secretion [4–6]. Notably, removing senescent astrocytes could prevent neurodegeneration and cognitive decline in aged mice [7–9]. Thus, targeting senescent astrocytes provides insights into therapeutic strategy for neurodegenerative diseases such as PD.

Mitochondrial dysfunction is a hallmark of aging and PD [10–12]. Damaged mitochondria releases reactive oxide species and mitochondrial DNA (mtDNA) into cytoplasm, which contributes to cell senescence and the progression of PD [13, 14]. As an outer mitochondrial membrane protein, mitofusin 2 (Mfn2) plays important roles in normalizing mitochondrial homeostasis through controlling the process of fission and fusion [15, 16]. Recently, Mfn2 has been demonstrated to participate in regulating cellular energy metabolism such as insulin resistance and glucose metabolism [17, 18]. However, whether Mfn2 is involved in astrocyte senescence remains unclear.

Metformin is a common hypoglycemic drug in clinic [19] and has recently been identified as a promising drug for anti-aging and neuroprotection in the brain [20–22]. However, the effects of metformin on astrocyte senescence in PD and the specific mechanism have not yet been documented. In the present study, we showed that metformin significantly delayed the astrocyte senescence in vitro and in vivo through astrocytic Mfn2-cGAS signal. Further study revealed that metformin targeted Mfn2 to normalize mitochondrial function to reduce mitochondrial DNA (mtDNA) release, thereby inhibiting the cGAS-STING signal activation, which contributed to delaying the astrocyte senescence and PD progression. Our study clarifies the molecular mechanism of astrocyte senescence in PD, and sheds light on the clinical decisions regarding the use of metformin in age-associated neurodegenerative diseases.

## Methods

### Experimental animals

Mice (C57Bl/6 background) were housed under standard laboratory conditions (22 ± 1 °C, 12 h light–dark cycle, food and water ad libitum) in the Animal Resource Centre of the Faculty of Medicine (Nanjing Medical University). All procedures concerning animal care and treatment were carried out in accordance with the guideline of the Institutional Animal Care and Use Committee (IACUC) of Nanjing Medical University.

### MPTP/p-induced PD mouse model

The MPTP protocol was carried out as described previously [23]. To evaluate the effect of metformin on cell senescence, the mice (male, 16–18 week) were injected subcutaneously with MPTP hydrochloride (25 mg/kg, Sigma-Aldrich, St. Louis, MO, USA) and then injected intraperitoneally with probenecid (250 mg/kg, Sigma, St. Louis, MO, USA) at 1 h interval for five consecutive days. One hour after first MPTP injection, mice were administered with metformin at 100 mg/kg/day for 2 week, Metformin was dissolved in drinking water and administered orally.

### Preparation of Adeno-associated virus (AAV) particles

AAV particles (AAV-PHP.eB-GFAP promoter-shRNA (cGAS)-EGFP, 5.5 × 10^12^ vg/mL) were purchased from Shanghai GeneChem Co. (Shanghai, China). Short hairpin sequences (cat# GOSV0290175, sh1: 5′- GGATTGAGCTACAAGAATA-3′, sh2: 5′- GCTGTAACACTTCTTATCA-3′, 5′- GCTGGTCTTGAACAAAGAGAT-3′) were cloned into the AAV.

### Stereotaxic injection

The stereotaxic injection was performed as previously described [24]. Under anesthesia, the 1.5 μL of AAV-PHP.eB-GFAP promoter-shRNA (cGAS)-EGFP (5.5 × 10^12^ vg/mL) was bilaterally injected into the substantia nigra pars compacta (SNpc) at a rate of 0.3 μL/min using the following coordinates: − 3.0 mm A/P, ± 1.3 mm M/L, and − 4.5 mm D/V from bregma.

### Behavioral analysis

The behavior test was assessed at 7 days after the final injection of MPTP as described previously [25]. All behavioral analyses were carried out during the light cycle. For the pole test, the mice were placed head upward on the top of a vertical wooden rough-surfaced pole (diameter 1 cm, height 50 cm). The total time until the mouse reached the floor with its four paws (T-total) and the time needed for the mouse to turn completely head downward (T-turn) were recorded. For the rotarod test, mice were accustomed to the apparatus for 2 days, and then placed on the rod and tested at 20 rpm for 5 min. The latency time that mouse stayed on the rod at 20 rpm was recorded. For the open field test, locomotor activity of mice was detected in an activity monitor. The mouse was placed into activity monitor chambers (50 cm × 50 cm × 50 cm) for 30 min, and the activities were recorded at 5-min intervals.

### Immunohistochemistry and immunofluorescence

Midbrain was cut into 30-μm slices to detect tyrosine hydroxylase (TH) using a freezing microtome (Leica M1950, Nussloch, Germany) as described previously [26], Brain slices were incubated with anti-TH antibody (T1299, 1:1000, Sigma, St Louis, MO, USA) overnight. After that, the brain slices were incubated with secondary antibodies for 1 h and then visualized by incubation in substrate-chromogen solution. The total number of TH-positive neurons in the SNpc were counted stereologically using the Optical Fractionator (Stereo Investigator 7, MBF bioscience, Williston, VT, USA).

For immunofluorescence staining, the slices or the astrocytes were incubated with anti-GFAP (MAB360, 1:1000 dilution, Millipore, Billerica, MA, USA), anti-TH (AB152, 1:2000 dilution, Sigma-Aldrich, USA), anti-IBA-1 (ab5076, 1:1000 dilution, Abcam), anti-lamin B1 (Abcam, ab16048, 1:400 dilution), anti-p16 (Santa Cruz Biotechnology, sc-56330, 1:200 dilution) or anti-cGAS (31659S, 1:400 dilution, Cell Signaling Technology, USA) overnight at 4 °C, followed by incubation in Alexa Fluor 555-conjugated antibody (Invitrogen, A21432; 1:1000) or Alexa Fluor 488-conjugated antibody (Invitrogen, A21202; 1:1000) for 1 h at 20 °C. The nuclei was stained with DAPI (P36931, Life Technologies). Images were obtained by a confocal microscope (Axiovert LSM510, Carl Zeiss Co., Germany) and then processed by Image J.

### Quantitative RT-PCR (qPCR)

Total RNA from these cultured astrocytes and SNpc tissue was extracted with Trizol reagent (Invitrogen, USA). Reverse transcription PCR was performed using a TAKARA PrimeScript RT reagent kit and qPCR was carried out in duplicate for each sample using a QuantiTect SYBR Green PCR kit (Qiagen, Germany) with an ABI 7300 Fast Real-Time PCR System (Applied Biosystems, Foster City, CA, USA). GAPDH was used as an internal control for the real-time PCR amplification. The sequences of primers used are as follows: IL-1β forward: TCATTGTGGCTGTGGAGAAG, reverse: AGGCCACAGGTATTTTGTCG. GAPDH forward: CAAAAGGGTCATCTCC, reverse: CCCCAGCATCAAAGGTG. p16^Ink4a^ forward: CGCTTCTCACCTCGCTTGT, reverse: TGACCAAGAACCTGCGACC. IL-1α forward: AGTCAACTCATTGGCGCTTG, reverse: GAGAGAGATGGTCAATGGCAGA. IL-6 forward: TCCTTCCTACCCCAATTTCCA, reverse: GTCTTGGTCCTTAGCCACTCC. MMP-3 forward: GTTCTGGGCTATACGAGGGC, reverse: TTCTTCACGGTTGCAGGGAG. MMP-9 forward: CGACTTTTGTGGTCTTCCCC, reverse: AGCGGTACAAGTATGCCTCTGATTTCCA.

### Culture and treatment of mouse primary astrocytes

Primary astrocyte was cultured as described previously [27]. The neonatal midbrain (P0-3) was trypsinized with 0.25% trypase at 37 °C for 10 min and then centrifuged for 5 min at 1000*g* centrifugation. The cells were resuspended in Dulbecco’s modified Eagle’s medium (DMEM)/Ham’s F12 medium containing 10% fetal bovine serum (FBS, GIBCO, Gaithersburg, MD, USA) and plated onto T-75 flasks at 50,000 cells/cm^2^. After 10 d, confluent mixed glial cultures were shaken at 220 rpm for 6 h at 37 °C to remove unwanted cell types (microglia, oligodendrocytes, neurons, and fibroblasts). The purity of astrocytes was > 95% as determined with GFAP immunocytochemistry. To induce premature senescence model, astrocytes were pretreated with metformin at the indicated concentration for 1 h and then stimulated with MPP^+^ (200 μM, Sigma, St. Louis, MO, USA) for 24 h or α-synuclein aggregate (α-Syn PFF, 1 μg/mL, ab218819, abcam, USA) for 48 h. To induce naturally senescence model, astrocytes were cultured for 40 days and then treated with metformin for 10 days in vitro.

### Cell transfection

For Mfn2 plasmid transfection, astrocytes were transfected with plasmids expressing Mfn2 (Hanbio Biotechnology Co., Ltd., Shanghai, China) in OPTI-MEM-reduced serum medium (Gibco, USA) using lipofectamine 3000 reagent (Invitrogen, Life Technologies) for 48 h according to the instructions provided.

### Mesencephalic primary neuron cultures and treatment

Mesencephalic primary neuron cultures were prepared from the ventral mesencephalic tissues of C57BL/6 mice on embryonic day 14/15 (E14/15). Briefly, mesencephalic cells were dissociated by trypsinization (0.25% trypsin and 0.02% EDTA in Ca^2+^- and Mg^2+^-free Hanks’ balanced salt solution) at 37 °C for 10 min, followed by gentle triturating in plating medium (h-DMEM supplemented with 10% fetal bovine serum and 10% horse serum). Cells were seeded onto poly-l-lysine-coated 24-well plates at a density of 2.5 × 10^5^ cells per cm^2^ and incubated at 37 °C in 5% CO_2_ atmosphere. After cell adherence, the medium was replaced by neurobasal medium supplemented with 2% B-27 (Gibco-BRL) and 0.5 mM l-glutamine (Sigma) and treated with 1 μM cytosine arabinoside (Sigma) for 24 h to inhibit glial cell proliferation. Half of the culture medium was replaced every 3.5 days. Cultures were used after 7 days in vitro. Astrocytic conditioned medium (ACM) was collected and centrifuged at 1000*g* for 5 min to remove debris and dead cells. Mesencephalic primary neurons were incubated with the supernatant mixed with neurobasal medium at a ratio of 1:2 for 24 h before immunocytochemical staining.

### Quantification of neuron count and neuronal processes

As described previously [28], mesencephalic primary neurons were stimulated with astrocytic conditioned medium (ACM) for 24 h. The cells were incubated with anti-MAP2 antibody (ab32454, Abcam, USA) at 4 °C overnight and then incubated with Alexa Fluor 488-conjugated antibody for 1 h at 20 °C. Neurons were counted and the total length of cell processes was quantitated using Image Pro Plus 5.1.

### Measurement of cytosolic mtDNA

Astrocytes were divided into two equal aliquots. One aliquot was used to extract whole-cell DNA. To isolate cytosolic DNA, the cells in other aliquot were resuspended in digitonin lysis buffer containing 150 mM NaCl, 50 mM HEPES pH 7.4, and 25 μg/mL digitonin (Sigma-Aldrich). The homogenates were incubated on a rotator for 10 min at 20 °C and then centrifuged at 16,000*g* for 25 min. The supernatant was used for qPCR. The copy number of mtDNA (Nd1) obtained from cytosolic extracts was normalized to the copy number of nuclear DNA (Tert) obtained from the whole-cell extracts. The sequences of primers used are as follows: Nd1 forward: CAAACACTTATTACAACCCAAGAACA, reverse: TCATATTATGGCTATGGGTCAGG. nDNA Tert forward: CTAGCTCATGTGTCAAGACCCTCTT, reverse: GCCAGCACGTTTCTCTCGTT.

### SA-β-gal staining

SA-β-gal staining was carried out with a β-galactosidase-based Senescence Cells Staining Kit (CS0030-1KT, Sigma-Aldrich, USA) according to the manufacturer’s instructions. Astrocytes were fixed in 4% paraformaldehyde for 30 min at 20 °C and then stained with the SA-β-gal staining solution overnight at 37 °C. The positive senescent astrocytes stained blue were counted.

### Measurement of mitochondrial superoxide

To detect superoxide in the mitochondria, astrocytes were stained with MitoSOX (M36008, Invitrogen, USA) at 2.5 μM for 30 min at 37 °C. After that, the cells were washed with PBS twice and then resuspended in cold PBS containing 1% FBS for flow cytometric analyses. Flow data were analyzed with the FCS Express software (Guava Easy Cyte™8, Millipore, USA).

For immunofluorescence, astrocytes were incubated with MitoSOX at 2.5 μM for 30 min at 37 °C and washed twice with PBS. DAPI visualizes nuclei. Images were acquired by a confocal microscope. Mitochondria-associated ROS levels were assessed by fluorescence intensity using Image J.

### Determination of mitochondrial membrane potential

To determine mitochondrial membrane potential, astrocytes were stained with 10 μg/mL JC1 (T-3168, Invitrogen, USA) for 30 min at 37 °C and then washed with PBS twice. After that the cells were resuspended in cold PBS containing 1% FBS for flow cytometric analyses. Flow data were analyzed with the FCS Express software (Guava Easy Cyte™8, Millipore, USA).

For immunofluorescence, astrocytes were incubated with JC1 for 30 min at 37 °C and washed twice with PBS. The cells then were incubated with DAPI for 10 min to visualize nuclei. Images were observed by a confocal microscope.

### Western blotting analysis

Brain tissues and astrocytes were lysed in RIPA lysis buffer. Each 30 μg protein was separated and then transferred onto PVDF membranes (IPVH00010, Millipore, MA, USA). The membranes were blocked with 10% milk for 1 h at 25 °C and then incubated with anti-p16 (ab211542, abcam, USA), anti-p-sting (72971S, Cell Signaling Technology, USA), anti-cGAS (31659S, Cell Signaling Technology, USA), anti-TH (MAB318, 1:1000, Millipore, Billerica, MA, USA), anti-Mfn2 (ab56889, abcam, USA), anti-p-AMPK (50081, Cell Signaling Technology, USA) and anti-β-actin (BM0627, Boster, Pleasanton, CA, USA) antibody overnight at 4 ◦C. Immuno-reactive bands were detected by ImageQuant™ LAS 4000 imaging system (GE Healthcare, Pittsburgh, PA, USA) and analyzed using ImageJ software.

### Statistical analysis

All data were analyzed using Prism7 software and were expressed as means ± SEM. The differences among different treatments and genotypes were assessed by one-way or two-way analysis of variance (ANOVA), followed by the Tukey’s post hoc test. The results were considered significant at p < 0.05.

## Results

### Metformin delays astrocyte senescence in MPTP-induced PD model

**Fig. 1:**
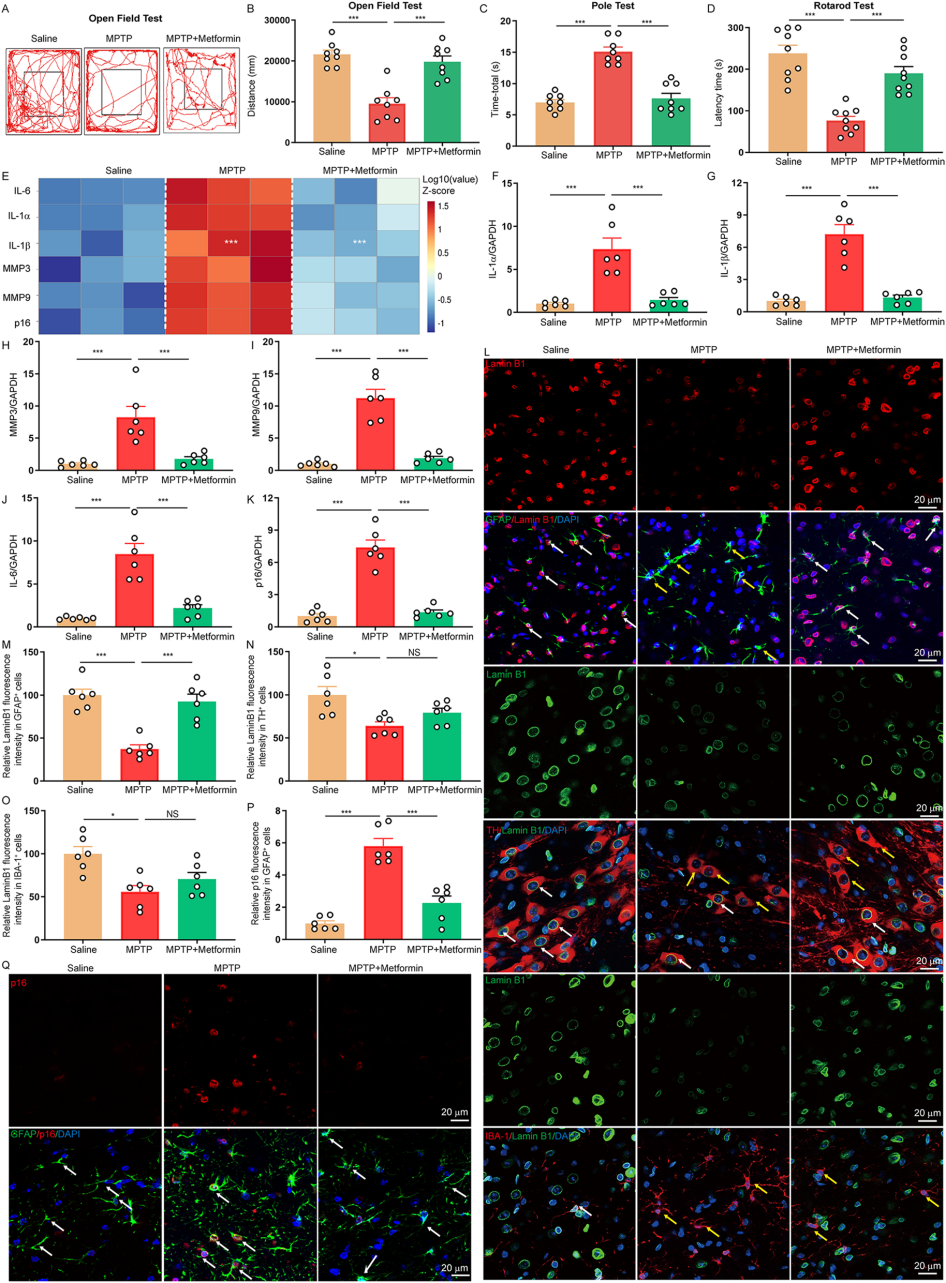
Metformin delays astrocyte senescence in MPTP-induced PD model. **A**, **B** Movement distance within 5 min was recorded by open field test (n = 8 animals for each group). **C** the time taken to descend a pole (Time-total) was recorded in pole test (n = 8 animals for each group). **D** Time on the rod was measured by the rotarod test (n = 9 animals for each group). **E** Heatmap of relative mRNA levels of SASP as indicated in SNpc (n = 3 animals for each group). **F–K** qPCR measurement of the levels of IL-1α (**F**), IL-1β (**G**), MMP3 (**H**), MMP9 (**I**), IL-6 (**J**) and p16^Ink4a^ (**K**) in the SNpc (n = 6 animals for each group). **L** Representative double-immunostaining for lamin B1 and astrocytic marker GFAP, DA neuron marker TH or microglia marker IBA-1 in the SNpc. DAPI stains nucleus (blue). White arrow: high level of lamin B1, yellow arrow: low level of lamin B1. **M–O** Quantification of lamin B1 immunofluorescence intensity in GFAP^+^ astrocytes (**M**), TH^+^ DA neuron (**N**), and IBA-1^+^ microglia (**O**) in the SNpc (n = 6 animals for each group). **P** Quantification of p16 immunofluorescence intensity in GFAP^+^ astrocytes in the SNpc (n = 6 animals for each group). **Q** Representative double-immunostaining for p16 (red) and astrocytic marker GFAP (green) in the SNpc. DAPI stains nucleus (blue). The data shown are the mean ± SEM. One-way ANOVA with Tukey’s post-hoc tests were used. *p < 0.05, ***p < 0.001, NS: no significant

The neuroprotective of metformin was evaluated in the MPTP model of PD. Compared to control mice, MPTP-treated mice showed a significant behavior impairment, which is prevented by metformin treatment (Fig. 1A–D). Cell senescence has suggested to be involved in PD progression [29]. To investigate the effects of metformin on cell senescence in PD, we measured the SASP factors secretion in the SNpc of PD mouse. qPCR analysis showed that the SASP factors, such as IL-6, IL-1α, IL-1β, MMP-3 and MMP9 were markedly increased in MPTP-treated mice and this increase was blunted by metformin (Fig. 1E–J). We also observed that metformin significantly reduced the senescence marker p16 ^INK4a^ expression in MPTP-treated mice (Fig. 1K), indicating that metformin inhibits cell senescence in PD. Subsequently, double immunostaining further confirmed that metformin evidently enhanced nuclear level of lamin B1 in MPTP-treated mice, mainly in GFAP^+^ astrocytes from the SNpc (Fig. 1L–O). In addition, metformin markedly diminished the level of senescence marker p16^INK4a^ in astrocytes of PD mice, detectable by immunostaining (Fig. 1P, Q). Collectively, the above findings demonstrate that metformin delays astrocyte senescence in MPTP-induced PD model.

### Metformin suppresses senescence of astrocytes in vitro

**Fig. 2:**
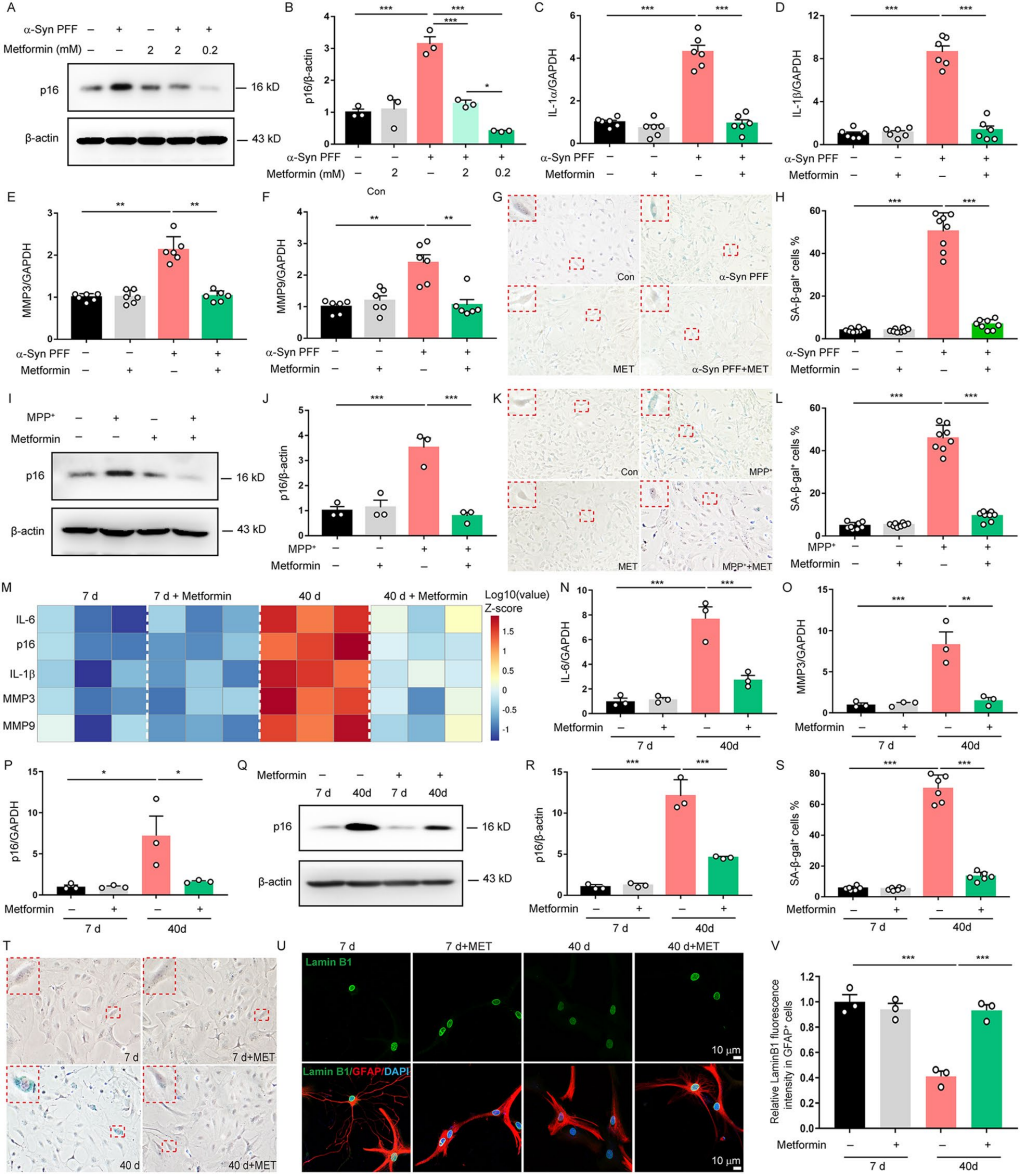
Metformin suppresses senescence of astrocytes in vitro. **A** Astrocytes were pretreated with metformin at indicated concentrations for 30 min and then stimulated with α-Syn PFF. Representative immunoblots of relative expression of p16 in astrocytes. **B** Quantification of relative expression of p16 in A (Three independent experiments). **C–F** qPCR measurement of the levels of IL-1α (**C**), IL-1β (**D**), MMP3 (**E**), and MMP9 (**F**) in astrocytes treated with metformin (0.2 mM) and α-Syn PFF (Six independent experiments). **G, H** Representative images of SA-β-gal staining and quantification of the percentage of SA-β-gal^+^ astrocytes over total astrocytes in astrocytes treated with metformin (0.2 mM) and α-Syn PFF (Three independent experiments). **I** Astrocytes were pretreated with metformin (0.2 mM) for 30 min and then stimulated with MPP^+^. Representative immunoblots of relative expression of p16 in astrocytes. **J** Quantification of relative expression of p16 in I (Three independent experiments). **K, L** Representative images of SA-β-gal staining and quantification of the percentage of SA-β-gal^+^ astrocytes over total astrocytes in astrocytes treated with metformin (0.2 mM) and MPP^+^ (Three independent experiments). **M** Astrocytes were cultured for 7 days or 40 days with or without metformin (0.2 mM). Heatmap of relative mRNA levels of SASP as indicated in astrocytes. **N–P** qPCR measurement of IL-6 (**N**), MMP3 (**O**), and p16 (**P**) mRNA expression in astrocytes (Three independent experiments). **Q, R** Representative immunoblots and quantification of relative expression of p16 in astrocytes (Three independent experiments). **S, T** Representative images of SA-β-gal staining and quantification of the percentage of SA-β-gal^+^ astrocytes over total astrocytes in astrocytes (Three independent experiments). **U, V** Immunofluorescence and quantification of lamin B1 in GFAP^+^ astrocytes (Three independent experiments). DAPI stains nucleus (blue). The data shown are the mean ± SEM. One-way ANOVA with Tukey’s post-hoc tests were used. *p < 0.05, **p < 0.01, ***p < 0.001

Next, we examined the direct effect of metformin on astrocyte senescence in vitro. To define it, we treated the astrocytes with α-Syn PFF, a pathological marker of PD, to induce premature senescence of astrocytes. α-Syn PFF significantly increased the expression of senescence marker p16^INK4a^ in astrocytes. Both supra-pharmacological plasma concentration (2 mM) and clinically relevant plasma concentration (0.2 mM) of metformin could inhibit the p16^INK4a^ expression in astrocytes (Fig. 2A, B). Since a more significant inhibitory effect of metformin was observed at the concentration of 0.2 mM, we used this concentration in the subsequent studies. Metformin treatment also reduced the secretion of SASP factors such as IL-1α, IL-1β, MMP-3 and MMP-9 induced by α-Syn PFF in astrocytes (Fig. 2C–F). SA-β-gal staining showed that metformin decreased the percentage of β-galactosidase positive cells (Fig. 2G, H). To confirm the effect of metformin on premature senescence of astrocytes, we also treated the astrocytes with MPP^+^, the active metabolite of MPTP significantly associated with PD. MPP^+^ induced astrocyte senescence phenotypes, including upregulation of senescence markers p16^INK4a^ and elevated senescence-associated β-galactosidase activity, which were indeed abrogated by metformin treatment (Fig. 2I–L).

To further verify the effect of metformin on astrocyte senescence, we cultured astrocytes in vitro for 40 days to induce naturally senescence of astrocytes. As shown in Fig. 2M–R, senescent astrocytes displayed elevated levels of p16^Ink4a^ mRNA and protein and increased production of SASP factors such as IL-6, IL-1α, MMP-9 and MMP-3, which were indeed delayed by metformin treatment (Additional file 1: Fig. S1). In addition, metformin treatment reduced the percentage of β-galactosidase positive cells and increased the expression of lamin B1 in senescent astrocytes, detectable by immunostaining (Fig. 2S–V). These findings indicate that metformin inhibits senescence of astrocytes in vitro.

### Metformin normalizes mitochondrial function to reduce the mtDNA release in senescent astrocytes by Mfn2

**Fig. 3:**
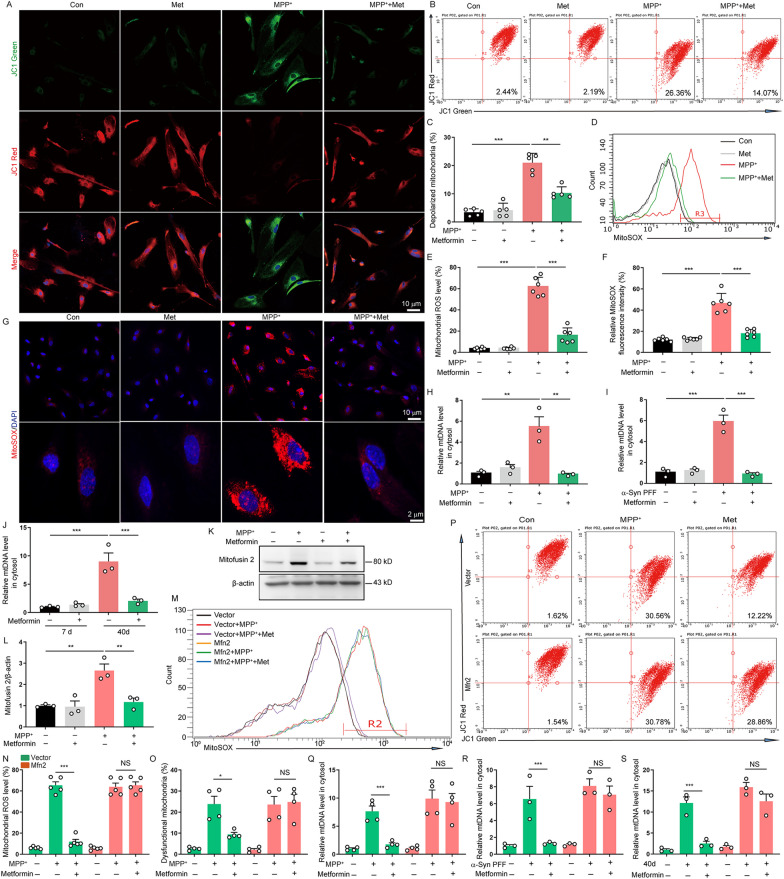
Metformin normalizes mitochondrial function in senescent astrocytes by Mfn2. **A** Astrocytes were pretreated with metformin (0.2 mM) for 30 min and then stimulated with MPP^+^. Representative images of JC-1 in astrocytes were analyzed using confocal microscopy (Three independent experiments). DAPI stains nucleus (blue). **B, C** Representative images and quantification of depolarized mitochondria analyzed by flow cytometry (Five independent experiments). **D, E** Representative images and quantification of mitochondrial ROS level analyzed by flow cytometry (Six independent experiments). **F, G** Representative images and quantification of mitochondrial ROS immunofluorescence intensity in immunofluorescence using ImageJ software (Six independent experiments). DAPI stains nucleus (blue). **H–J** qPCR measurement of mtDNA in cytosol of astrocytes pretreated with metformin (0.2 mM) and then stimulated with MPP^+^ (**H**), α-Syn PFF (**I**) or cultured for 40 days (**J**) (Three independent experiments). **K, L** Representative immunoblots and quantification of relative expression of Mfn2 in astrocytes treated with metformin (0.2 mM) and MPP^+^ (Three independent experiments). **M, N** Astrocytes were transfected with empty vector (pHBLV-CMV-MCS-3FLAG-EF1-ZsGreen-T2A-PURO) or Mfn2 plasmid for 48 h and then treated with metformin (0.2 mM) and MPP^+^. Representative images and quantification of mitochondrial ROS level analyzed by flow cytometry (Five independent experiments). **O, P** Representative images and quantification of dysfunctional mitochondria analyzed by flow cytometry (Four independent experiments). **Q–S** qPCR measurement of mtDNA in cytosol of astrocytes stimulated with MPP^+^ (**Q**), α-Syn PFF (**R**) or cultured for 40 days (**S**) (Three independent experiments). The data shown are the mean ± SEM. One-way ANOVA with Tukey’s post-hoc tests were used (**C, E, F, H–J, L**). Two-way ANOVA with Tukey’s post-hoc tests were used (**N**, **O**, **Q–S**). **p < 0.01, ***p < 0.001, NS: no significant

As AMPK is potential effector of metformin [30], we then examined whether metformin suppressed astrocytes senescence through AMPK. We treated astrocytes with AMPK inhibitor compound C and found that the inhibitory effects of metformin on astrocytes senescence were not completely abrogated by compound C (Additional file 1: Fig. S2). These results suggest that metformin delays astrocytes senescence in an AMPK-independent manner. Accumulation of defective mitochondria has been reported to play crucial roles in cell senescence [31]. To dissect the underlying mechanism of metformin inhibiting the senescence of astrocytes, we then quantified mitochondrial membrane potential in astrocytes with a fluorescence probe JC-1 assay system. Mitochondrial membrane potential disruption was detected in MPP^+^-induced senescent astrocytes and this disruption was restored by metformin treatment, as measured by immunofluorescence and flow cytometric analyses (Fig. 3A–C). In addition, robust mitochondrial ROS production was observed in MPP^+^-induced senescent astrocytes and this increase was also reversed by metformin treatment, as measured by mitochondrial superoxide indicator MitoSOX (Fig. 3D–G). Meanwhile, qPCR analysis showed that mtDNA was obviously increased in both MPP^+^ and α-Syn PFF-induced premature senescent astrocytes and long-term culture-induced naturally senescent astrocytes, and this increase was abrogated by metformin (Fig. 3H–J). More importantly, the level of Mfn2 was significantly enhanced in MPP^+^-induced premature senescent astrocytes and this enhancement was restored by metformin (Fig. 3K–L). Mfn2 plays a crucial role in mitochondrial homeostasis [32]. To investigate whether metformin normalized mitochondrial function through Mfn2 in senescent astrocytes, we re-expressed Mfn2 in astrocytes by transfecting the Mfn2 gene. The enforced expression of Mfn2 canceled the protective effects of metformin on mitochondrial function in astrocyte, including mitochondrial swelling, mitochondrial membrane potential disruption, enhanced mitochondrial ROS production and increased mtDNA release (Fig. 3M–S, Additional file 1: Fig. S3). These findings indicate that metformin improves mitochondrial function to decrease mtDNA release in astrocytes through Mfn2.

### Metformin inactivates the cGAS-STING signal in senescent astrocytes in vitro and in vivo

**Fig. 4:**
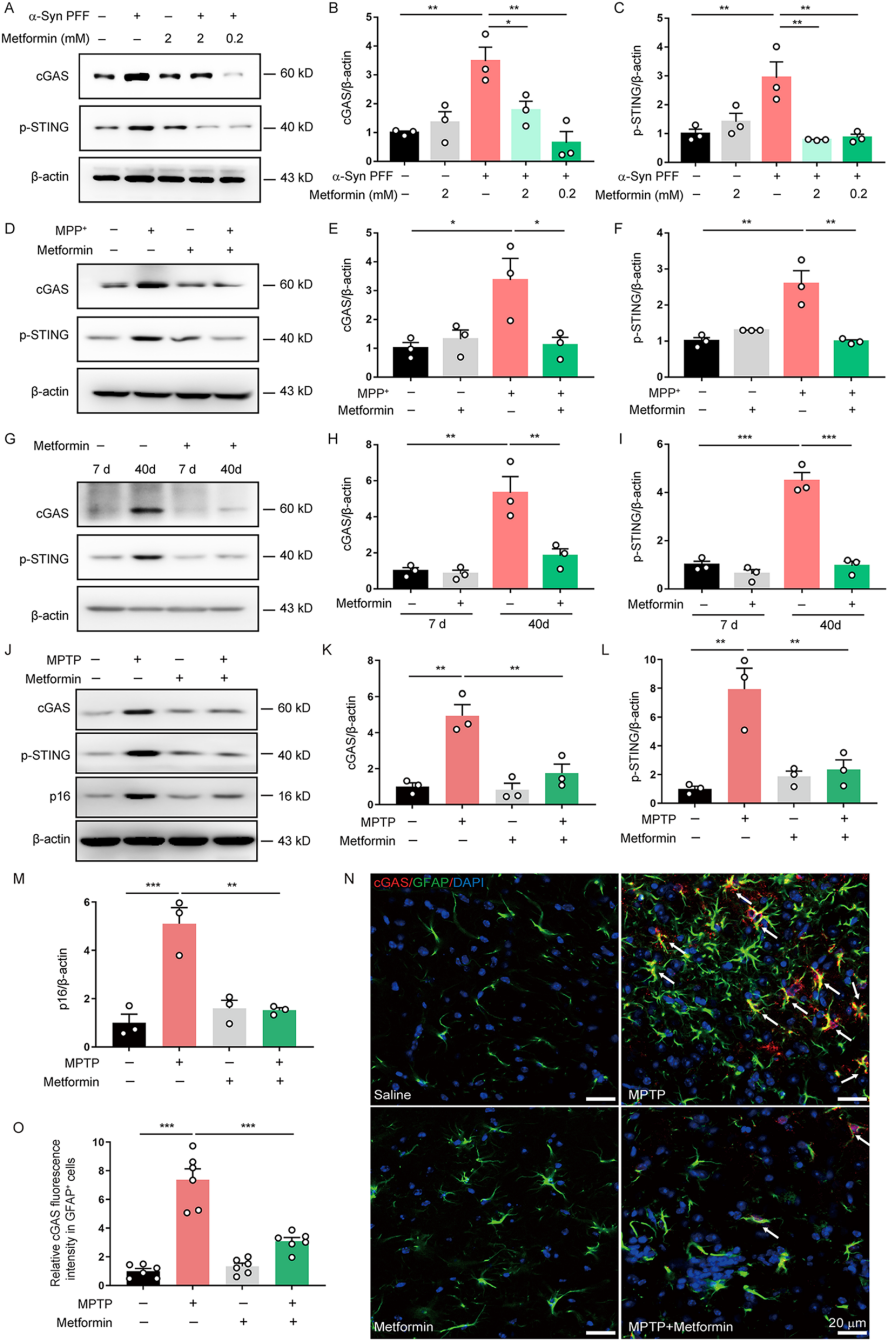
Metformin inactivates the cGAS-STING signal in senescent astrocytes in vitro and in vivo. **A–C** Astrocytes were pretreated with metformin at indicated concentrations for 30 min and then stimulated with α-Syn PFF. Representative immunoblots (**A**) and quantification of relative expression of cGAS (**B**) and p-STING (**C**) in astrocytes (Three independent experiments). **D–F,** Astrocytes were pretreated with metformin (0.2 mM) for 30 min and then stimulated with MPP^+^. Representative immunoblots (**D**) and quantification of relative expression of cGAS (**E**) and p-STING (**F**) in astrocytes (Three independent experiments). **G–I,** Astrocytes were pretreated with metformin (0.2 mM) and then cultured for 40 days. Representative immunoblots (**G**) and quantification of relative expression of cGAS (**H**) and p-STING (**I**) in astrocytes (Three independent experiments). **J–M** Representative immunoblots (**J**) and quantification of relative expression of cGAS (**K**), p-STING (**L**) and p16 (**M**) in the SNpc from MPTP-treated mice (n = 3 animals for each group). **N** Representative double-immunostaining for cGAS (red) and astrocytic marker GFAP (green) in the SNpc. DAPI stains nucleus (blue). **O** Quantification of the cGAS level in GFAP^+^ astrocytes from SNpc (n = 6 animals for each group). The data shown are the mean ± SEM. One-way ANOVA with Tukey’s post-hoc tests were used. *p < 0.05, **p < 0.01, ***p < 0.001

As cGAS-STING signal is activated by recognizing cytosolic DNA and involved in cell senescence [33], we next determined the effect of metformin on the activation of the cGAS-STING signal in astrocytes. We found that the expression of cGAS and p-STING was obviously increased in α-Syn PFF-induced senescent astrocytes compared with non-senescent astrocytes. But, this increase was abolished by metformin treatment (Fig. 4A–C), which was consistent with its inhibitory effect on astrocyte senescence. Since astrocyte senescence can be triggered by MPP^+^, we then investigated the effects of metformin on cGAS-STING signal activation in MPP^+^-induced senescent astrocytes. Our results showed that metformin also suppressed cGAS expression and STING phosphorylation induced by MPP^+^ (Fig. 4D, F). Long-term culture-induced naturally senescent astrocytes were further verify the effects of metformin on cGAS-STING signal activation. As shown in Fig. 4G–I**,** the activation of cGAS-STING signal was significantly enhanced in naturally senescent astrocytes compared with young astrocytes and this enhancement was reversed by metformin treatment. These findings demonstrate that metformin suppresses the cGAS-STING signal activation in senescent astrocytes.

Following these findings in astrocytes in vitro, we performed a series of animal experiments to determine whether metformin can affect the activation of cGAS-STING signal in mice. Similar to the results obtained in vitro, MPTP-treated mice showed a significant activation of cGAS-STING signal, which is companied by elevated expression of senescence marker p16^INK4a^. Metformin treatment markedly inhibited the activation of cGAS-STING signal and reduced the expression of p16^INK4a^ in the SNpc of MPTP-treated mice, detected by western blot analysis (Fig. 4J–M). Subsequently, double immunostaining further confirmed that expression of cGAS was evidently increased in MPTP-treated mice, mainly in GFAP^+^ astrocytes from the SNpc, and this increase also suppressed by metformin treatment (Fig. 4N, O**)**. These findings indicate that metformin inactivates the astrocytic cGAS-STING signal in MPTP-induced mouse model of PD.

### Metformin delays astrocytes senescence by blocking astrocytic Mfn2-cGAS

**Fig. 5:**
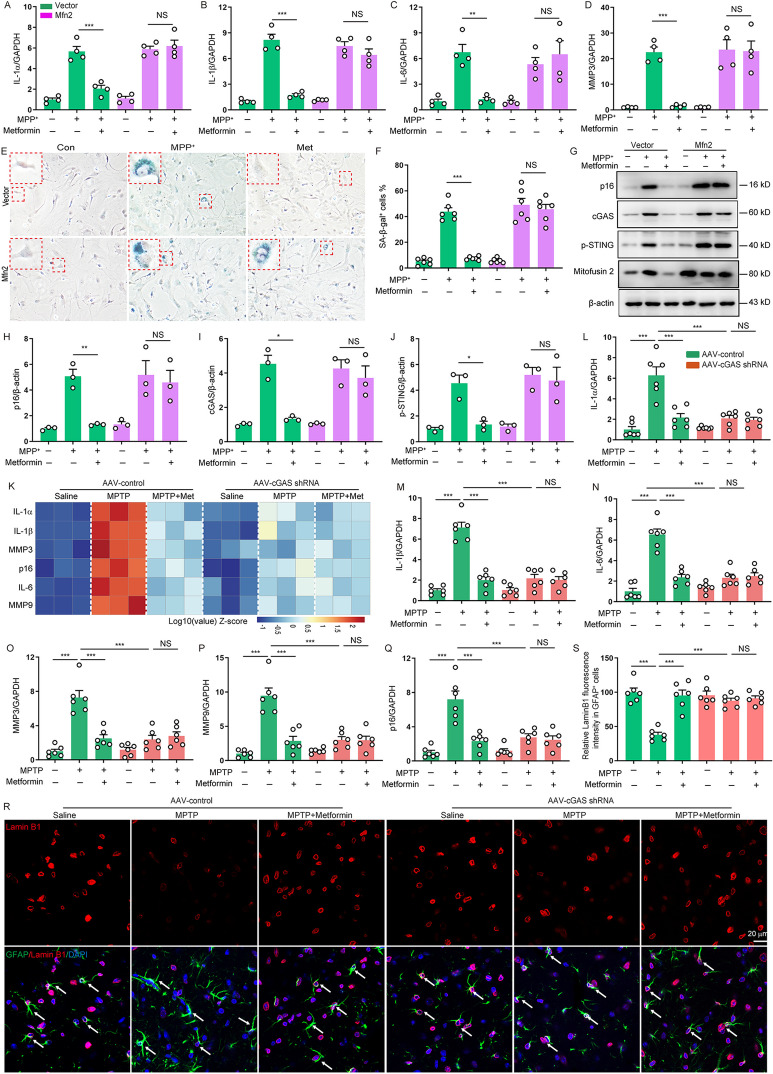
Metformin delays astrocytes senescence by blocking astrocytic Mfn2-cGAS. **A–D** Astrocytes were transfected with vector or Mfn2 plasmid for 48 h and then treated with metformin (0.2 mM) and MPP^+^. qPCR measurement of the levels of IL-1α (**A**), IL-1β (**B**), IL-6 (**C**), and MMP3 (**D**) in astrocytes (Four independent experiments). **E**, **F** Representative images of SA-β-gal staining and quantification of the percentage of SA-β-gal^+^ astrocytes over total astrocytes in astrocytes (Three independent experiments). DAPI stains nucleus (blue). **G–J** Representative immunoblots (**G**) and quantification of relative expression of p16 (**H**), cGAS (**I**) and p-STING (**J**) in astrocytes (Three independent experiments). **K** Heatmap of relative mRNA levels of SASP as indicated in SNpc (n = 3 animals for each group). **L–Q** qPCR measurement of the levels of IL-1α (**L**), IL-1β (**M**), IL-6 (**N**), MMP3 (**O**), MMP9 (**P**), and p16^Ink4a^ (**Q**) in the SNpc (n = 6 animals for each group). **R** Representative double-immunostaining for lamin B1 and astrocytic marker GFAP in the SNpc. DAPI stains nucleus (blue). **S** Quantification of lamin B1 immunofluorescence intensity in GFAP^+^ astrocytes in the SNpc (n = 6 animals for each group). The data shown are the mean ± SEM. Two-way ANOVA with Tukey’s post-hoc tests were used. **p < 0.01, ***p < 0.001, NS: no significant

To determine whether metformin suppressed astrocytes senescence through Mfn2, we upregulated the Mfn2 expression by transfecting the Mfn2 gene. We found that metformin significantly decreased the secretion of SASP factors including IL-1α, IL-1β, IL-6 and MMP-3, diminished senescence-associated β-galactosidase activity and reduced the expression of p16 in vector-transfected astrocytes, but this effect was not observed in Mfn2-transfected astrocytes (Fig. 5A–H, Additional file 1: Fig. S4). Meanwhile, we also observed that Mfn2 overexpression reversed the inhibitory effects of metformin on the activation of cGAS-STING in senescent astrocytes **(**Fig. 5I, J**)**. These results suggest that metformin delays astrocytes senescence through astrocytic Mfn2-cGAS. To further confirm whether metformin inhibits astrocytes senescence via astrocytic cGAS in vivo, we injected AAV carrying GFAP-promoter-cGAS-shRNA to mouse SNpc regions to specifically reduce astrocytic cGAS expression (Additional file 1: Fig. S5). As expected, metformin treatment efficiently reduced SASP factors such as IL-6, IL-1α, IL-1β, MMP3 and MMP9, diminished the p16 ^INK4a^ expression, and enhanced nuclear level of lamin B1 in astrocytes of PD mice, but this effect was not observed in astrocytic cGAS deficient mice (Fig. 5K–S). These data indicate that metformin suppresses astrocytes senescence in MPTP-induced PD model by blocking astrocytic Mfn2-cGAS.

### Metformin ameliorates PD-like pathology in MPTP-induced PD model via delay of cGAS-mediated astrocytes senescence

**Fig. 6:**
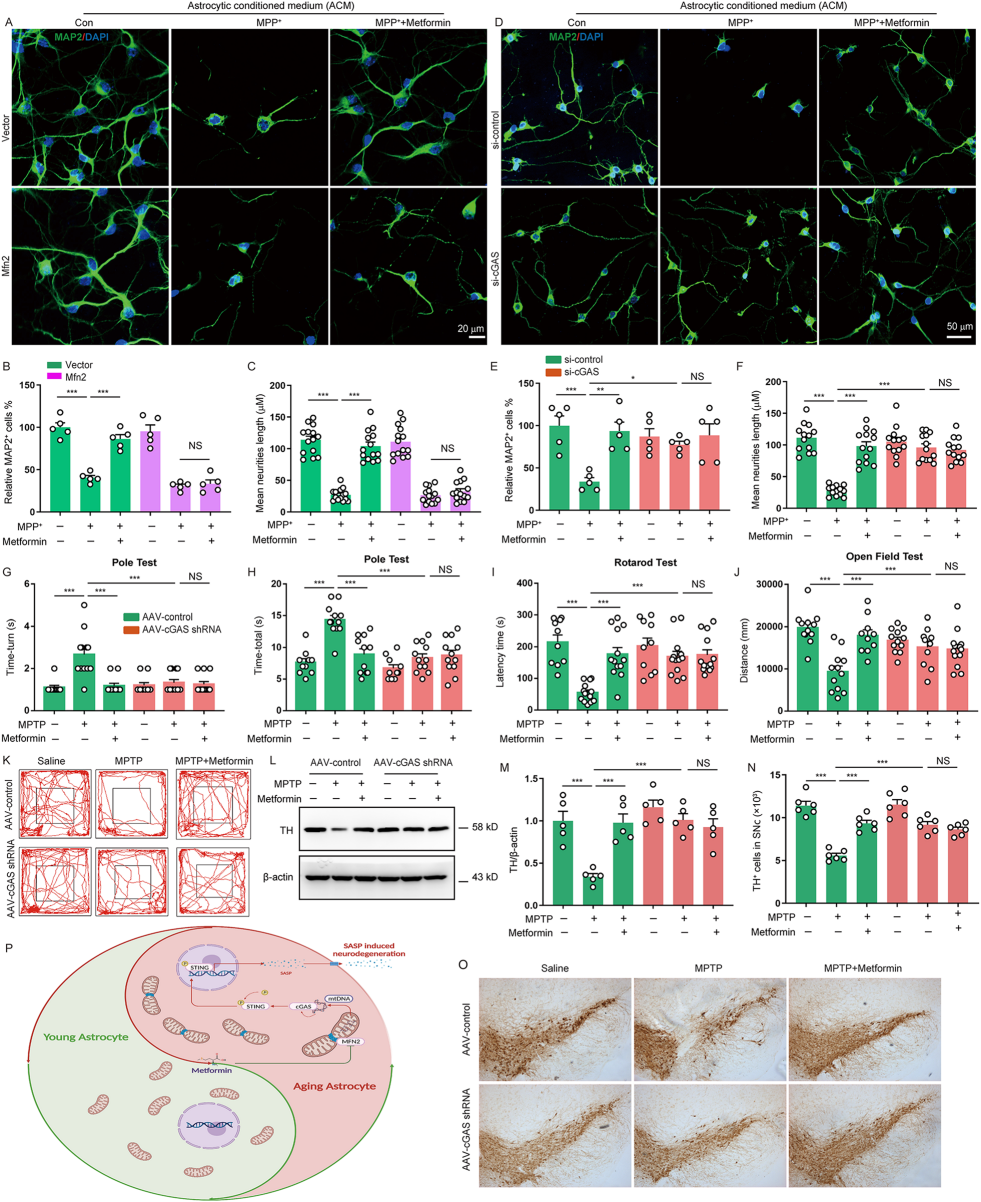
Metformin ameliorates PD-like pathology in MPTP-induced PD model via delay of cGAS-mediated astrocytes senescence. **A** Mesencephalic primary neurons were treated with astrocytic conditioned medium (ACM) from astrocytes transfected with Mfn2 plasmid and then stimulated with metformin (0.2 mM) and MPP^+^. Representative pictures of MAP2 (green) immunostaining. DAPI stains nucleus (blue). **B, C** Quantification of relative the number of neurons (**B**) and mean total neuritis length (**C**, Three independent experiments). **D** Mesencephalic primary neurons were treated with astrocytic conditioned medium (ACM) from astrocytes transfected with cGAS siRNA (si-cGAS) and then stimulated with metformin (0.2 mM) and MPP^+^. Representative pictures of MAP2 (green) immunostaining. DAPI stains nucleus (blue). **E, F** Quantification of relative the number of neurons (**E**) and mean total neuritis length (**F**, four independent experiments). **G, H** the time taken to turn around (Time-turn) and descend a pole (Time-total) was recorded in pole test (n = 9–12 animals for each group). **I** Time on the rod was measured by the rotarod test (n = 10–13 animals for each group). **J, K** Movement distance within 5 min was recorded by open field test (n = 10–13 animals for each group). **L, M** Representative immunoblots (**L**) and quantification of relative expression of TH (**M**) in the SNpc (n = 5 animals for each group). **N** Microphotographs of TH-positive neurons in the SNpc. **O** Stereological counts of TH-positive neurons in the SNpc (n = 6 animals for each group). **P** Proposed model depicting metformin inhibits astrocyte senescence via Mfn2-mtDNA-cGAS-STUNG signal in PD. The data shown are the mean ± SEM. Two-way ANOVA with Tukey’s post-hoc tests were used. **p < 0.01, ***p < 0.001, NS: no significant

Senescent astrocytes secrete SASP factors, which has been proved to play a crucial role in progression of PD [34]. We first determined whether metformin affects neurodegeneration in vitro. Murine mesencephalic primary neurons were stimulated with ACM from metformin-treated astrocytes. MAP2 immunostaining revealed that metformin significantly increased both the number of neurons and the length of neuronal processes, but this effect was not observed in Mfn2-transfected astrocytes and in cGAS-deficient astrocytes (Fig. 6A–F). Subsequently, we then evaluated whether metformin had any effect on Parkinsonism in mice. We found that metformin significantly improved the behavior deficit of MPTP-treated mice, but this improvement was not observed in astrocytic cGAS deficient mice (Fig. 6G, K). In addition, metformin treatment noticeably increased TH protein level in MPTP-treated PD mice, but this increase was rescued by astrocytic cGAS deletion (Fig. 6L, M). Moreover, metformin treatment obviously reversed the loss of DA neurons in PD model mice, as defined by TH immunostaining in the SNpc, but this effect was abolished in astrocytic cGAS deficient mice (Fig. 6N, O). These data demonstrate that metformin alleviates PD-like pathology in mice via delay of cGAS-mediated astrocytes senescence.

## Discussion

The most critical finding presented here is that metformin inhibits astrocyte senescence. Metformin is the common first-line drug to reduce blood glucose levels in patients with type 2 diabetes mellitus [35]. Recently, metformin also exerts neuroprotective activities [36] and extends lifespan [37]. But, the effects of metformin on astrocyte senescence remains unclear in PD. In the present study, we found that metformin decreased SA-β-gal activity, downregulated senescence marker p16^Ink4a^ expression, reduced several SASP factors production, and increased nuclear level of lamin B1 in both long-term culture-induced naturally senescence model and MPP^+^/α-Syn PFF-induced premature senescence model. More importantly, we also demonstrated that metformin treatment reduced the expression of p16^Ink4a^, increased the nuclear lamin B1 level and decreased the secretion of several SASP factors in astrocytes in the MPTP-induced PD model mice. These data indicate that metformin could delay astrocyte senescence in vitro and in PD model mice. Interestingly, we found that metformin restored Lamin B1 levels specifically in astrocytes, not in DA neurons or microglia, indicating metformin mainly inhibits astrocyte senescence, rather than delay of senescence of DA neurons and microglia in MPTP-induced PD model. It is plausible that metformin exerts different effects on distinct cell subtypes during different stages of PD progression. At the onset or early stages of PD, metformin mainly prevents oxidative stress-induced injury in DA neurons and suppresses inflammation in microglia, while at the middle or late stages of PD, it delays cell senescence in astrocytes. Therefore, long-term use of metformin is beneficial for delaying PD progression.

Our study further elucidates the molecular mechanism underlying by which metformin inhibits astrocyte senescence. Among the numerous potential targets of metformin identified, AMPK has been recognized as the central stage [38]. But we found that metformin (0.2 mM) still could suppress astrocyte senescence when AMPK inhibitor was used, indicating that metformin at this dose delays astrocytes senescence in an AMPK-independent manner. Emerging evidence has revealed that mitochondrial dysfunction are believed to be involved in cell senescence [39]. The cellular mitochondrial population undergoes repeated cycles of fission and fusion to maintain its integrity, as well as overall cellular homeostasis. Mfn2 plays a crucial role in regulating mitochondrial fusion, cellular metabolism, and various other cell function [40]. Several previous studies have reported that Mfn2 was downregulated in microglia after spinal cord injury and then leads to an imbalance in mitochondrial fusion and division, inducing the release of mtDNA, which mediates the activation of the cGAS-STING signaling pathway, and overexpression of MFN2 may alleviate inflammatory response and the symptoms of disease [41, 42]. On the contrary, in the situation of aging, previous results indicate that MFN2 expression was elevated in both human and rat chondrocytes during aging and osteoarthritis, and overexpression of MFN2 exacerbated inflammation and osteoarthritis progress [43]. This is consistent with our results. Here, we also found that Mfn2 expression was increased in MPP^+^-induced senescent astrocytes, companied by mtDNA release and cGAS-STING activation. Conflicting results may be due to differences in disease models, cell types, stress patterns or mechanisms. Either high level Mfn2-induced hyperfusion or low level of Mfn2-induced excessive fission may have negative impact on cellular health under pathological conditions. Therefore, Mfn2 expression (fusion-fission balance) needs to be fine-tuned to maintain proper biological homeostasis. Interestingly, we unveiled that metformin down-regulated the increased Mfn2 expression and then normalized mitochondrial function to reduce mtDNA release. This led to the inactivation of cGAS-STING signal, further inhibiting astrocyte senescence (Fig. 6P). Furthermore, Mfn2 overexpression reversed the effects of metformin, including improvement of mitochondrial function, decrease in mtDNA release, inactivation of cGAS-STING, and delay of astrocytes senescence. More importantly, astrocytic cGAS deletion abolished the suppressive effects of metformin on astrocyte senescence in PD model mice. Collectively, the current work reveals that metformin delays astrocyte senescence through Mfn2-mtDNA-cGAS.

Accumulating evidence demonstrates that astrocyte senescence plays a detrimental role the progression of PD and ageing [44, 45]. Thus, targeting astrocyte senescence might be an effective approach for treating PD and aged-associated neurodegenerative diseases. In the present study, we found that metformin noticeably alleviated the behavior dysfunction and DA neuron loss in PD model mice, companied by reduced the accumulation of senescent astrocytes and inactivation of astrocytic cGAS-STING in SNpc. cGAS-STING, components of the innate immune system, is involved in neuroinflammation and cellular senescence [46–48]. Here, we observed that astrocytic cGAS deletion reversed the protective effects of metformin on astrocyte senescence and neurodegeneration in PD model mice. Additionally, the ACM from metformin-treated astrocytes results in more negligible DA neuron injury, but this effect was abolished by cGAS deletion. This further supported the crucial role of cGAS-mediated astrocytes senescence in PD pathogenesis. These finding demonstrate that metformin effectively prevents DA degeneration in mouse PD model via suppression of cGAS-mediated astrocytes senescence.

## Conclusions

Our study reveals that metformin prevents DA neurodegeneration in PD via delay of astrocyte senescence by blocking astrocytic Mfn2-mtDNA-cGAS signal. This work indicates that metformin is a potential therapeutic agent for age-associated neurodegenerative disease such as PD.

### Supplementary Information

**Additional file 1. Fig. S1** Metformin suppresses SASP secretion in senescent astrocytes. **Fig. S2** Metformin suppresses senescence of astrocytes in an AMPK-independent manner. **Fig. S3** Mfn2 overexpression cancels the protective effects of metformin on mitochondrial swelling in astrocytes. **Fig. S4** The inhibitory effect of metformin on SASP factors depends on the level of Mfn2 in astrocytes. **Fig. S5** AAV-mediated cGAS shRNA is expressed in astrocytes in the SNpc.

## Data Availability

The datasets generated during and/or analyzed during the current study are available from the corresponding author on reasonable request.

## Abbreviations

PD: Parkinson’s disease
SNpc: Substantia nigra compacta
MPP^+^: 1-Methyl-4-phenylpyridinium
DA: Dopaminergic
MPTP: 1-Methyl-4-phenyl-1,2,3,6-tetrahydropyridine
TH: Tyrosine hydroxylase
GFAP: Glial fibrillary acidic protein
SASP: Senescence-associated secretory phenotype
SA-β-Gal: Senescence-associated β-galactosidase
ROS: Reactive oxygen species
mtDNA: Mitochondrial DNA
Mfn2: Mitofusin 2
cGAS: Cyclic GMP-AMP synthase
STING: Stimulator of interferon genes
