# Supplementary material for: Metformin normalizes mitochondrial function to delay astrocyte senescence in a mouse model of Parkinson’s disease through Mfn2-cGAS signaling
Source: J Neuroinflammation. 2024 Apr 2;21:81. doi: 10.1186/s12974-024-03072-0 (PMC10986112; doi:10.1186/s12974-024-03072-0)
Supplement: Supplementary file 1 — Additional file 1. Fig. S1 Metformin suppresses SASP secretion in senescent astrocytes. Fig. S2 Metformin suppresses senescence of astrocytes in an AMPK-independent manner. Fig. S3 Mfn2 overexpression cancels the protective effects of metformin on mitochondrial swelling in astrocytes. Fig. S4 The inhibitory effect of metformin on SASP factors depends on the level of Mfn2 in astrocytes. Fig. S5 AAV-mediated cGAS shRNA is expressed in astrocytes in the SNpc. [file 12974_2024_3072_MOESM1_ESM.docx]

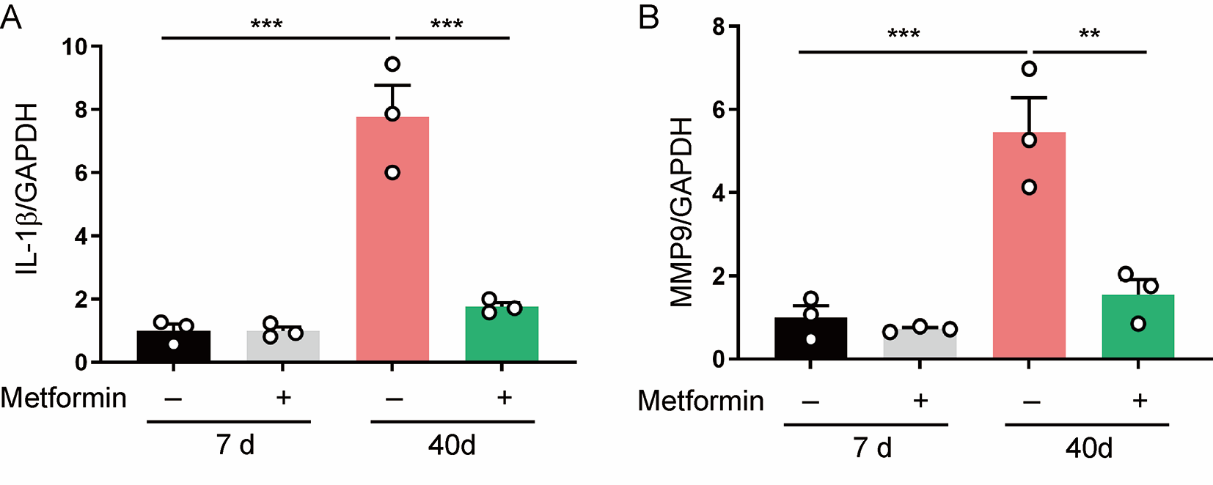


**Fig. S1 Metformin suppresses SASP secretion in senescent astrocytes.**

**A-B**, Astrocytes were treated with metformin (0.2 mM) and cultured for 7 days or 40 days. qPCR measurement of IL-β (A) and MMP9 (B) mRNA expression in astrocytes (Three independent experiments). The data shown are the mean ± SEM. One-way ANOVA with Tukey’s post-hoc tests were used. ^**^p<0.01, ^***^p<0.001.


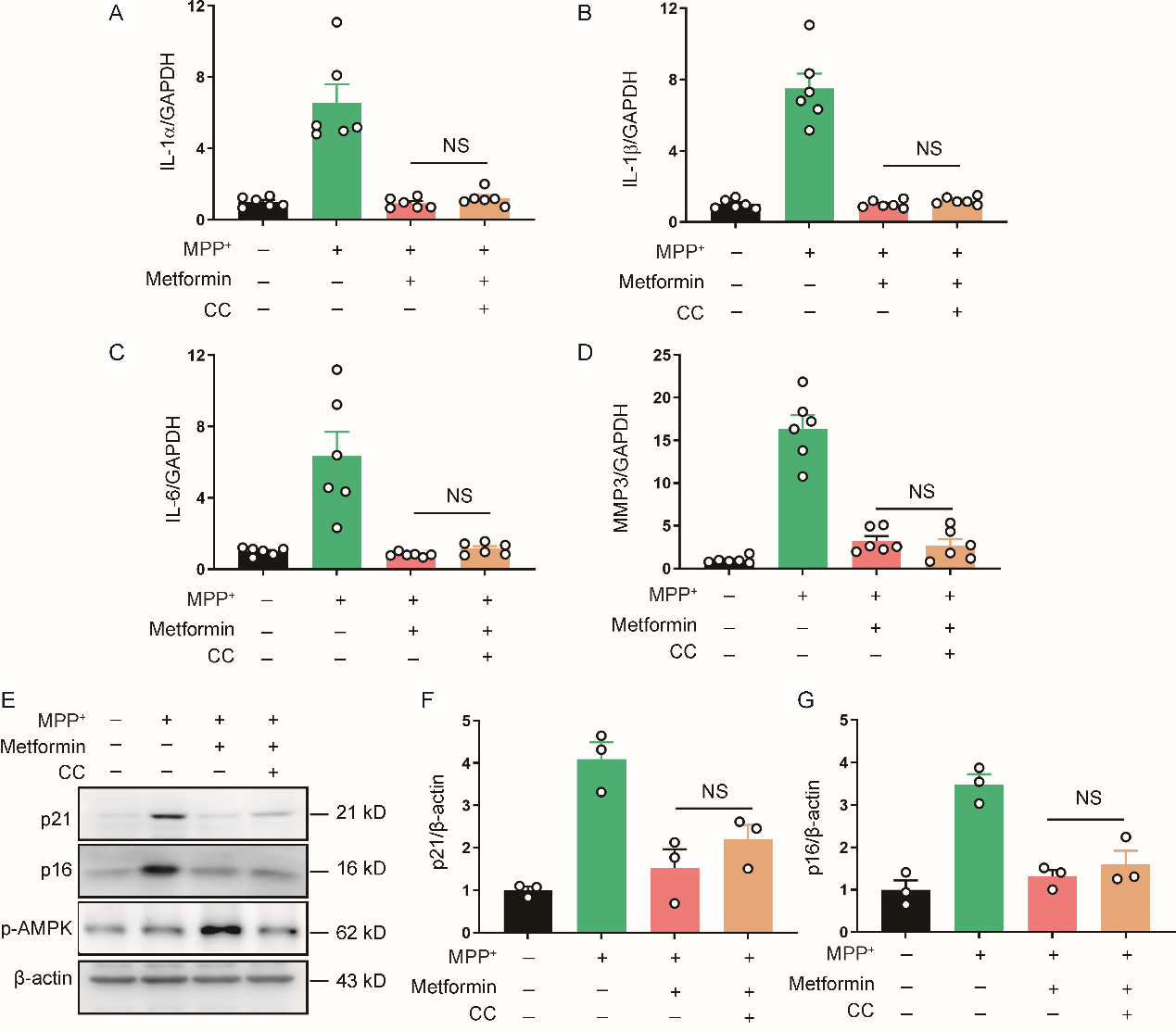


**Fig. S2 Metformin suppresses senescence of astrocytes in an AMPK-independent manner**

**A-D**, Astrocytes were treated with AMPK inhibitor compound C (CC) for 30 min and then stimulated with metformin (0.2 mM) and MPP^+^. qPCR measurement of IL-1α (A), IL-1β (B), IL-6 (C) and MMP3 (D) in astrocytes (Six independent experiments). **E-G,** Representative immunoblots (E) and quantification of relative expression of p21 (F), p16 (G) and p-AMPK in astrocytes (Three independent experiments). The data shown are the mean ± SEM. One-way ANOVA with Tukey’s post-hoc tests were used. NS: no significant.


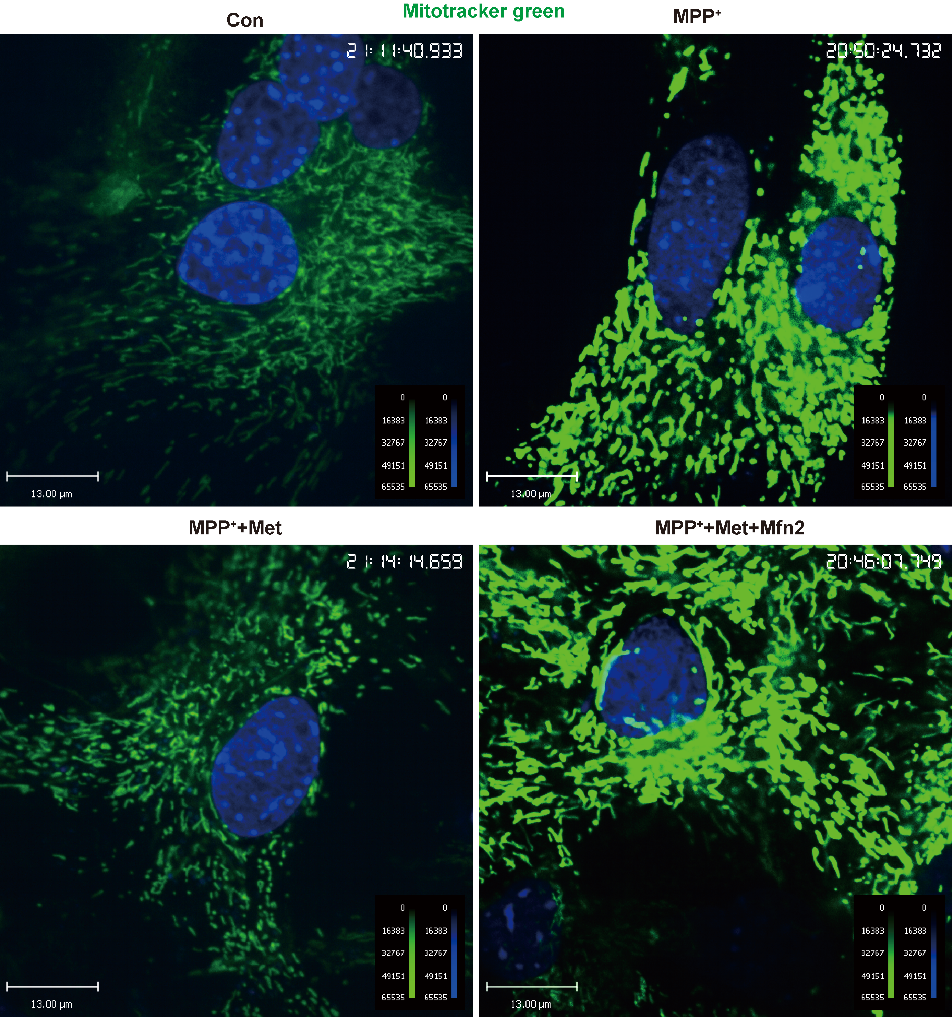


**Fig. S3 Mfn2 overexpression cancels the protective effects of metformin on** **mitochondrial swelling in astrocytes.**

Astrocytes were transfected with Mfn2 plasmid for 48 h and then treated with metformin (0.2 mM) and MPP^+^. Confocal microscopy of morphological changes in mitochondria monitored by staining with Mitotracker green.


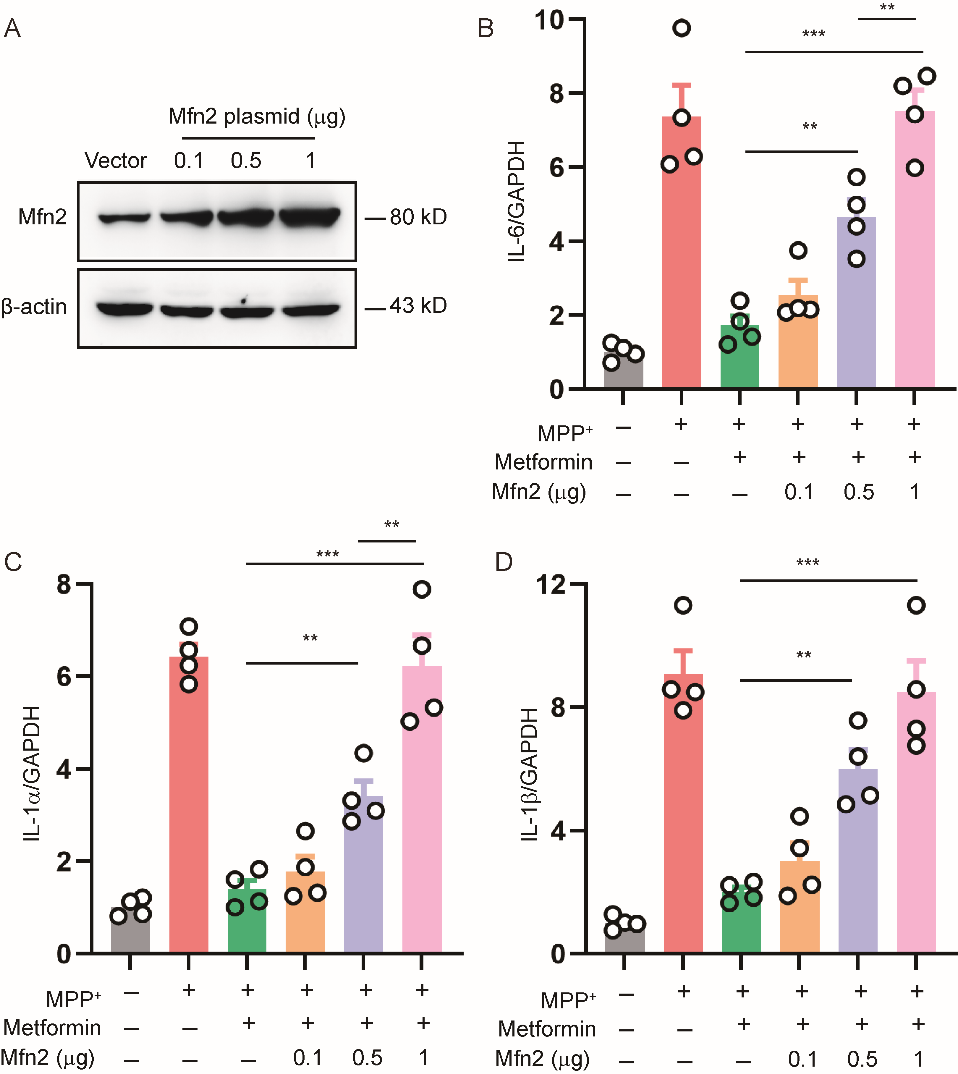


**Fig. S4 The inhibitory effect of metformin on SASP factors depends on the level of Mfn2 in astrocytes.**

**A,** Representative immunoblots of Mfn2 expression in astrocytes transfected with empty vector or Mfn2 plasmid as indicated concentration for 48h. **B-D**, Astrocytes were transfected with Mfn2 plasmid as indicated concentration for 48 h and then treated with metformin (0.2 mM) and MPP^+^. qPCR measurement of the levels of IL-6 (B), IL-1α (C) and IL-1β (D) in astrocytes


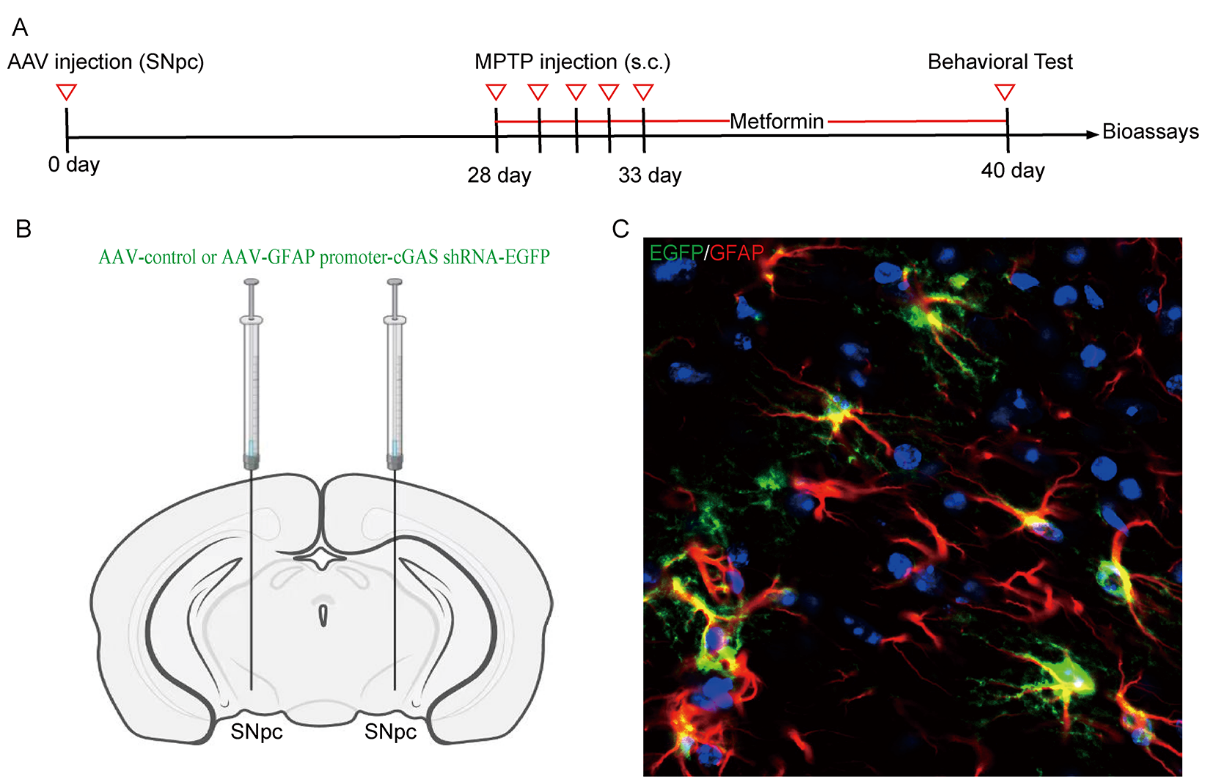


**Fig. S5 AAV-mediated cGAS shRNA is expressed in astrocytes in the SNpc.**

**A-B,** Diagram of the experimental design. **C,** IHC results confirm that AAV-cGAS shRNA (EGFP) is expressed, mainly in GFAP^+^ astrocytes (red).
